# A novel type of *N*-acetylglutamate synthase is involved in the first step of arginine biosynthesis in *Corynebacterium glutamicum*

**DOI:** 10.1186/1471-2164-14-713

**Published:** 2013-10-18

**Authors:** Kathrin Petri, Frederik Walter, Marcus Persicke, Christian Rückert, Jörn Kalinowski

**Affiliations:** 1 Microbial Genomics and Biotechnology, Center for Biotechnology, Bielefeld University, Universitätsstraße 27 33615 Bielefeld, Germany

**Keywords:** *Corynebacterium glutamicum*, *N*-acetylglutamate synthase, NAGS, Arginine biosynthesis, ArgA, HPLC-ESI-qTOF

## Abstract

**Background:**

Arginine biosynthesis in *Corynebacterium glutamicum* consists of eight enzymatic steps, starting with acetylation of glutamate, catalysed by *N*-acetylglutamate synthase (NAGS). There are different kinds of known NAGSs, for example, “classical” ArgA, bifunctional ArgJ, ArgO, and S-NAGS. However, since *C. glutamicum* possesses a monofunctional ArgJ, which catalyses only the fifth step of the arginine biosynthesis pathway, glutamate must be acetylated by an as of yet unknown NAGS gene.

**Results:**

Arginine biosynthesis was investigated by metabolome profiling using defined gene deletion mutants that were expected to accumulate corresponding intracellular metabolites. HPLC-ESI-qTOF analyses gave detailed insights into arginine metabolism by detecting six out of seven intermediates of arginine biosynthesis. Accumulation of *N*-acetylglutamate in all mutants was a further confirmation of the unknown NAGS activity. To elucidate the identity of this gene, a genomic library of *C. glutamicum* was created and used to complement an *Escherichia coli* Δ*argA* mutant. The plasmid identified, which allowed functional complementation, contained part of gene *cg3035*, which contains an acetyltransferase domain in its amino acid sequence. Deletion of *cg3035* in the *C. glutamicum* genome led to a partial auxotrophy for arginine. Heterologous overexpression of the entire *cg3035* gene verified its ability to complement the *E. coli* Δ*argA* mutant *in vivo* and homologous overexpression led to a significantly higher intracellular *N*-acetylglutamate pool. Enzyme assays confirmed the *N*-acetylglutamate synthase activity of Cg3035 *in vitro*. However, the amino acid sequence of Cg3035 revealed no similarities to members of known NAGS gene families.

**Conclusions:**

The *N*-acetylglutamate synthase Cg3035 is able to catalyse the first step of arginine biosynthesis in *C. glutamicum*. It represents a novel class of NAGS genes apparently present only in bacteria of the suborder *Corynebacterineae*, comprising amongst others the genera *Corynebacterium*, *Mycobacterium*, and *Nocardia*. Therefore, the name C-NAGS (*Corynebacterineae*-type NAGS) is proposed for this new family.

## Background

**Figure 1:**
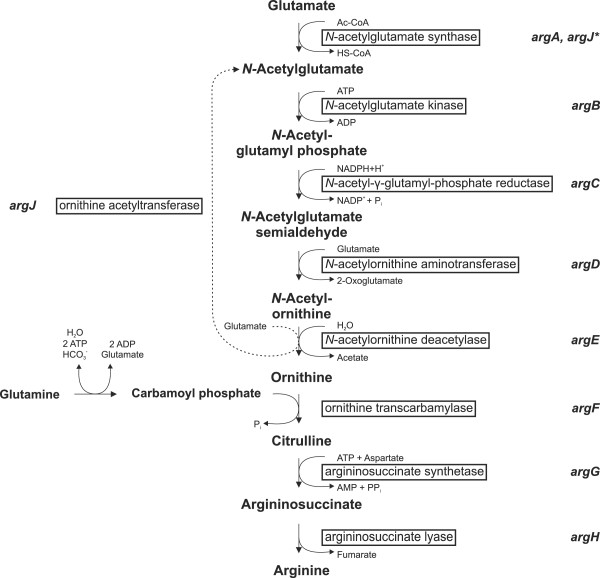
**General pathway of arginine biosynthesis in prokaryotes including two known routes for removal of the acetyl group.** Vertical arrows represent the linear pathway, whereas the alternative cyclic pathway in which the acetyl group is recycled by ornithine acetyltransferase (encoded by *argJ*) is indicated by a dashed arrow. Intermediates and immediate precursors are given in bold letters, enzymes are in boxes. ArgJ* designates bifunctional proteins. Abbreviations: HS-CoA = coenzyme A; Ac-CoA = acetyl-CoA; (P)P_i_ = (pyro)phosphate; HCO_3_- = bicarbonate.

In prokaryotes, biosynthesis of arginine from glutamate (Figure 1) is carried out by a series of eight enzymatic reactions initiated by acetylation of glutamate, a reaction catalysed by *N*-acetylglutamate synthase (NAGS). This first step prevents glutamate from cyclisation and its further use in proline biosynthesis [1]. After metabolisation of *N*-acetylglutamate, biosynthesis proceeds via three enzymatic steps which form further acetylated intermediates, until the acetyl group is removed in the fifth step of this process. The resulting ornithine is carbamoylated to citrulline. Addition of aspartate leads to *N*-argininosuccinate, which is finally converted to l-arginine [2].

Enzymes catalysing the formation of *N*-acetylglutamate in prokaryotes exhibit a high degree of diversity. *N*-acetylglutamate synthase activity was first discovered in *Escherichia coli*[3]. Here, the two-domain protein ArgA contains a carbamate kinase domain at its N-terminus that is homologous to the next enzyme in arginine biosynthesis, *N*-acetylglutamate kinase (NAGK, ArgB) [4]. The *N*-acetylglutamate synthase domain, as well as an acetyl-CoA binding region, is located at the C-terminus of ArgA. This domain is related to the large family of GCN5-related acetyltransferases (GNAT) [2]. ArgA from *E. coli* is strongly feedback-inhibited by l-arginine, leading to 50% inhibition at 0.02 mM [5]. Coenzyme A and *N*-acetylglutamate also inhibit the activity of this enzyme: 2.5 mM CoA or 25 mM *N*-acetylglutamate both result in 50% inhibition of ArgA [6]. In *E. coli*, removal of the acetyl group is catalysed by ArgE (*N*-acetylornithine deacetylase) during the fifth step of arginine biosynthesis, which results in a linear pathway [7].

For the acetylated intermediates, the majority of prokaryotes have a more efficient cyclic pathway in which ArgJ (ornithine acetyltransferase, OAT) catalyses the acetylation of glutamate [8]. ArgJ operates as a bifunctional protein and uses both substrates, acetyl-CoA and *N*-acetylornithine, thus, this enzyme exhibits both NAGS and OAT activity [9]. In this case, NAGS activity is essential for priming arginine biosynthesis, but it has only an anaplerotic function while most *N*-acetylglutamate is produced by ArgJ using l-glutamate and *N*-acetylornithine as substrates. The acetyl group is recycled via transacetylation of l-glutamate. Therefore the latter reaction is generally preferred, explaining the low abundance of NAGS enzymes in organisms exhibiting OAT [1].

Regulation of this metabolic pathway is generally achieved through feedback inhibition of the second enzyme in the pathway, *N*-acetylglutamate kinase (ArgB) by l-arginine [10] and/ or by feedback inhibition of ArgJ by l-ornithine [8].

In most bacteria, either a bifunctional ArgJ or ArgA is responsible for formation of *N*-acetylglutamate. However, there also exist some organisms such as *Neisseria gonorrhoeae*[11] or *Bacillus stearothermophilus*[12] exhibiting both a bifunctional OAT as well as a NAGS gene. There are also OATs which are unable to use acetyl-CoA as substrate and are, therefore, designated as ‘monofunctional’. An additional NAGS gene is needed in this case, however, in organisms such as *Streptomyces coelicolor* or *Thermus thermophilus,* both encoding a monofunctional ArgJ, no orthologue of ArgA can be identified by sequence similarity searches [13]. In these organisms, the glutamate acetylation mechanism remains unclear, however, in 2005, Errey *et al.* discovered a novel ArgA protein in *Mycobacterium tuberculosis*, containing only a single domain and consisting of 174 amino acids [13]. This “short NAGS” version (S-NAGS) is a putative GNAT-related enzyme and was later also found in other genera including *Thermus* and *Streptomyces.* The proteins of this family possess only 150 to 180 amino acids, similar to the length of the C-terminal acetyl-CoA domain of classical NAGS, however, are able to complement *E. coli argA* mutants [14].

In addition to single-domain S-NAGSs, another group of proteins exists in which a S-NAGS domain is fused with an *N*-argininosuccinase (ArgH) domain, the protein that catalyses the last step of the l-arginine biosynthesis pathway. The *argH(A)* genes were found mainly in marine bacteria of the *Alteromonas-Vibrio* group [14,15]. It is speculated that these S-NAGSs always require a complex formation with a protein partner providing an efficient glutamate binding site [2].

*Corynebacterium glutamicum* is a natural producer of l-glutamate, a precursor of l-arginine and, accordingly, its arginine biosynthesis has been the subject of intense research. The earliest studies of this organism were performed in 1958 by Udaka and Kinoshita, who analysed the metabolic pathway of l-ornithine, an intermediate of the arginine pathway. The authors recognised that acetylated compounds form a cycle of reactions, in which the acetyl group is recycled by generation of *N*-acetylglutamate [16]. Hence, it was concluded that *C. glutamicum* exhibits an OAT activity catalysed by ArgJ. In 1996, Sakanyan *et al*. discovered the monofunctional action of ArgJ by revealing its ability to complement *E. coli argE* but not *argA* mutants. By performing enzyme inhibition tests, they detected no influence on OAT activity by l-arginine, however, a product inhibition was shown when 5 mM l-ornithine was added to the ArgJ enzyme [8].

Nevertheless, ArgJ lacks NAGS activity and so investigations to discover a glutamate acetylase responsible for the first step in arginine biosynthesis were performed. Recently, Hwang and Cho [17], who searched for *C. glutamicum N*-acetyltransferase genes complementing an auxotrophic ∆*argJ* mutant, claimed that Cg1722 (NCgl1469) demonstrates NAGS activity and that its overexpression leads to an increase in ornithine production. But shortly thereafter Kind *et al.*[18] revealed that Cg1722 acetylates diaminopentane (cadavarine) instead of glutamate, so that the NAGS gene of *C. glutamicum* is still unknown.

In the work described here, we analysed arginine synthesis in *C. glutamicum* by individually mutating single genes known to be involved in this synthesis pathway and determining the patterns of accumulated metabolic intermediates. In a complementation approach, we identified a *C. glutamicum* gene able to complement an arginine-auxotrophic *E. coli argA* mutant. Enzyme activity tests as well as metabolic profiling demonstrated that this gene is the functional analogue of ArgA in *C. glutamicum*.

## Results

Arginine biosynthesis of *C. glutamicum* was an integral part of a number of previous studies which have elucidated detailed knowledge of the genetic and enzymatic organisation of this pathway [8], its regulation by the transcriptional regulator ArgR, as well as the feedback inhibition of ArgB by the end-product of the pathway, l-arginine [19]. In order to complement the existing understanding of this pathway, we chose to characterise the metabolic intermediates of arginine metabolism using defined individual knock-out mutants of all known arginine biosynthesis genes located in the arginine operon [8].

**Figure 2:**
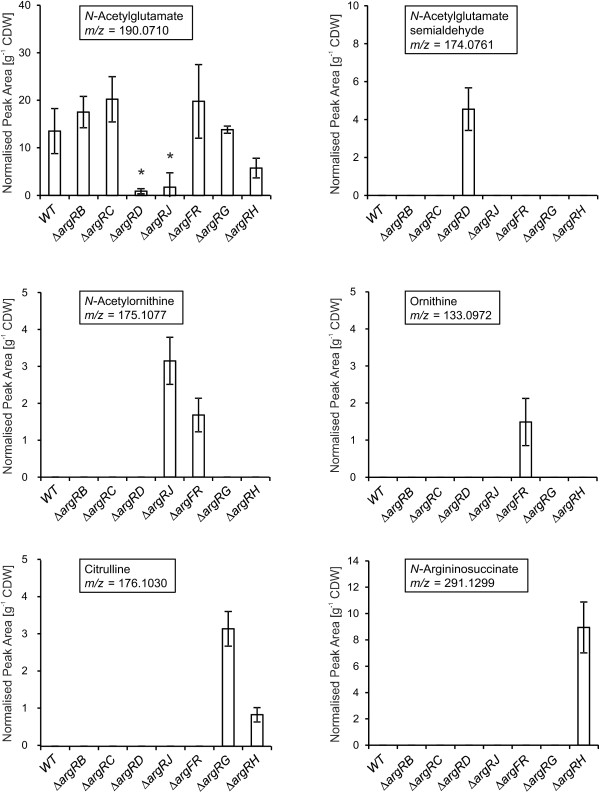
**Bar charts of normalised peak areas of six intermediates of arginine biosynthesis after HPLC-ESI-qTOF analysis.***C. glutamicum* ATCC 13032 (WT) and seven double deletion mutants were cultivated with l-arginine until exponential phase. Then l-arginine was removed and cells were further incubated to accumulate intracellular metabolites. The boxes in each diagram indicate the respective intracellular compound and its mass-to-charge ratio. Peak detection and integration was performed on base peak chromatograms (BPC) of m/z-values of [M + H]^+^ ions. Values that are significantly different from the wildtype level (Student’s T-test p < 0.05) are indicated by an asterisk. Error bars represent standard deviations of four biological replicates.

For this systematic metabolic study, auxotrophic mutants for each arginine biosynthetic gene of the *arg*-operon with an additional deletion of the transcriptional regulator of arginine biosynthesis (e.g. Δ*argRC*, Δ*argRJ*) were constructed. The knockout of the transcriptional repressor ArgR led to transcriptional activation of the ArgR regulon, consisting of the *arg*-operon, its sub-operon *argGH,* and the *carABpyrF*-operon as demonstrated by microarray hybridization analysis (Additional file 1). To ensure equal conditions, *C. glutamicum* ATCC 13032 and all mutants were cultivated in minimal media supplemented with l-arginine. By washing exponentially grown cells and transferring them into unsupplemented CGXII-medium, it is expected that auxotrophic strains starve and growth is arrested while the direct substrate of the removed enzyme should, therefore, accumulate, as has been shown in previous studies [20,21]. For detection of arginine biosynthesis intermediates, hydrophilic extracts were analysed by HPLC-ESI-qTOF in positive ionisation mode. Mean values for normalised peak areas of identified [M + H]^+^ ions of *N*-acetylglutamate, *N*-acetylglutamate semialdehyde, *N*-acetylornithine, ornithine, citrulline and *N*-argininosuccinate are depicted in Figure 2. *N*-acetylglutamyl phosphate was not detectable in any sample examined, which could be due to insufficient ionisation of this compound in positive mode. Arginine was as well not detected in any sample as was expected for deletion mutants. For *N*-acetylglutamate semialdehyde, no reference substance was available. Therefore, the respective [M + H]^+^ ion (m/z = 174.0761) was fragmented by applying automated MS/MS. The resulting spectrum was compared to a theoretical fragmentation pattern. The main fragment ion (m/z = 114.0550) can be explained by consecutive neutral losses of one molecule H_2_0 and one acetyl group (C_2_H_2_O). After revision of full-scan MS data, [M + H-H_2_O]^+^ ion (m/z = 156.0655) and [M + H-H_2_0-C_2_H_2_O]^+^ ion (m/z = 114.0517) could be observed in chromatograms of ∆*argRD,* that co-eluted with [M + H]^+^ ion of *N*-acetylglutamate semialdehyde (Additional file 2).

Comparison of metabolite profiles of different arginine auxotrophic strains revealed that the intracellular intermediates *N*-acetylglutamate semialdehyde, ornithine and *N*-argininosuccinate were only accumulated in their corresponding mutant, namely in Δ*argRD,* Δ*argRF,* or Δ*argRH,* respectively. However, citrulline was detected in samples of strains Δ*argRG*, and Δ*argRH*. The latter finding could indicate a backlog from *N*-argininosuccinate to citrulline or, alternatively, a low conversion rate of argininosuccinate synthetase. *N*-Acetylornithine was also found in two of the mutants. A high pool size of this compound was observed in ∆*argRJ* and a lower pool size in ∆*argRF,* which again might imply a backlog. The accumulation of *N*-acetylornithine in ∆*argRJ* provides clear *in vivo* evidence for a NAGS activity in *C. glutamicum*, catalysed by at least one other enzyme than ArgJ. Former experiments performed by Sakanyan *et al.* in 1996 revealed that ArgJ of *C. glutamicum* only exhibited monofunctional enzyme activity *in vitro* and *in vivo*[8].

*N*-Acetylglutamate was the only metabolite intermediate detectable in the wildtype and in all mutants. As in the ∆*argRJ* mutant*,* the *N*-acetylglutamate pool was significantly reduced in the ∆*argRD* strain, most probably due to the lack of substrate for transacetylation by ArgJ. However, the relatively high *N*-acetylglutamate pool in the ∆*argRC* mutant is not directly explained by this.

### Discovering a gene responsible for the first step of arginine biosynthesis

For identification of a gene encoding the missing *N*-acetylglutamate synthase in *C. glutamicum* ATCC 13032, an *E. coli* JM109 ∆*argA* mutant was constructed by using Red/ET recombination. After successful integration of the gene deletion cassette, the selection marker was removed by using FLP recombination. The constructed mutant was tested on M9 minimal medium and no growth was observed without l-arginine supplementation. Furthermore, arginine auxotrophy of this mutant was not complemented by ArgJ from *C. glutamicum*, as described in literature [8].

**Figure 3:**
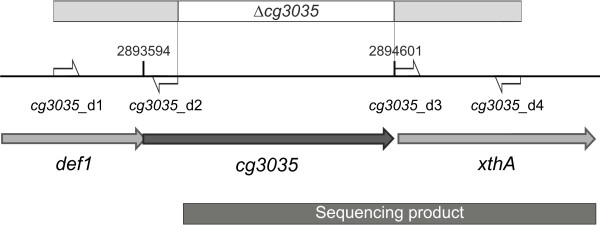
**Genomic map of the chromosomal region of *C. glutamicum *carrying *cg3035*.***Cg3035* is indicated as dark grey arrow, adjacent ORFs as light grey arrows. The cloned region of the complementation plasmid is shown as dark grey box. Also depicted are the binding positions of primers (small open arrows) used to generate the deletion construct (light grey boxes). The deleted region is depicted as empty box.

To discover a gene able to complement the auxotrophic *E. coli* ∆*argA* mutant, a genomic library of *C. glutamicum* ATCC 13032 was created. The procedure to create a DNA library and its cloning in the shuttle expression vector pZ8-2 are described in the Methods section. *E. coli* ∆*argA* mutants were transformed with the plasmid library. After 48 h of growth, a single colony was obtained on minimal medium plates. The plasmid was isolated from this clone and the DNA insert was sequenced. Analysis of the nucleotide sequence revealed a 1631 bp large DNA fragment of the *C. glutamicum* ATCC 13032 genome [22] (Figure 3)*.* The insert contained the 3’ part of gene *cg3035*, encoding a putative *N*-acetylglutamate synthase and the complete *xthA* gene (*cg3036*) coding for exodeoxyribonuclease III. Since a protein with *N*-acetylglutamate synthase activity (NAGS) was searched for, Cg3035 with its GNAT-domain (glutamate *N*-acetyltransferase domain) was the promising candidate. Although the DNA fragment carried only around 800 bp of the 1008 bp *cg3035-*gene, a Pfam database search (UniProt entry Q8NM40_CORGL) disclosed that the acetyltransferase domain of this protein is fully encoded on the cloned DNA fragment.

The genomic organisation of *cg3035* revealed an operon structure together with *cg3034*[23], coding for *def1*, a putative peptide deformylase, since coding sequences of these two genes share an 11 bp long overlap. Moreover, this implies a co-regulation of both proteins by translational coupling. The gene product of *cg3035* is 37.2 kDa in size and possesses an acetyltransf_1 (PF00583)-domain catalysing transfer of an acetyl group to its substrate [24]. A coenzyme A binding pocket is located upstream of the acetyltransferase domain. Nevertheless, an experimental validation of Cg3035 protein function was necessary.

### Validating the gene function of *cg3035* by heterologous complementation, gene deletion, metabolome analyses, and enzyme assays

The complete open reading frame of *cg3035* was amplified from *C. glutamicum* WT genome by PCR and cloned into pZ8-1 together with a consensus ribosome-binding site (RBS) of highly expressed genes [25]. *ArgA* from *E. coli* was treated similarly to serve as positive control. The resulting plasmids (pZ8-1::*argA*, pZ8-1::*cg3035*) were transformed into *E. coli* Δ*argA*. As expected, *argA* from *E. coli* allowed growth of arginine-auxotrophic mutant on minimal medium plates without l-arginine supplementation. Growth on minimal medium was also restored by *cg3035*, showing a functional complementation.

To test whether *cg3035* is involved in arginine biosynthesis in *C. glutamicum*, growth of the deletion mutant ∆*cg3035* was analysed on solid minimal medium without l-arginine supplementation and compared to the *C. glutamicum* wildtype (Additional file 3). After 48 h at 30°C the mutant ∆*cg3035* exhibits poor growth on unsupplemented MM1 plates in comparison to the *C. glutamicum* wildtype, indicating a bradytrophy of the mutant due to arginine limitation. Therefore, Cg3035 is required for full activity of arginine biosynthesis in *C. glutamicum*, but not essential.

**Figure 4:**
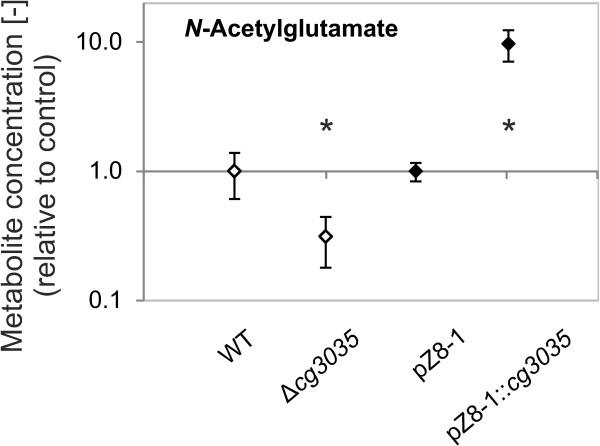
**Diagram of normalised peak areas of *N*-acetylglutamate in different *C. glutamicum *strains.** Hydrophilic metabolites were extracted from *C. glutamicum* ATCC 13032 (WT), ∆*cg3035* as well as WT pZ8-1 (empty vector) and WT pZ8-1::*cg3035.* Peak detection and integration was performed on base peak chromatograms of m/z-values of [M + H]^+^ ions. Error bars represent standard deviations of four biological replicates. Values that are significantly different from the wildtype level (Student’s T-test p < 0.01) are indicated by an asterisk.

To characterise the function of Cg3035 *in vivo*, we performed additional metabolic profiling experiments, in which we compared samples of the deletion mutant ∆*cg3035* and the overexpression strain WT pZ8-1::*cg3035* with regard to potential acetylation products formed by Cg3035. Samples of *C. glutamicum* ATCC 13032 (WT) and *C. glutamicum* WT pZ8-1 served as controls, respectively. Relative quantification of metabolites revealed a strong decrease of the *N*-acetylglutamate pool (fold change 0.31) in the deletion mutant ∆*cg3035* in comparison to the wildtype. In accordance to that, overexpression of Cg3035 led to an almost tenfold (fold change 9.69) increase of *N*-acetylglutamate (Figure 4), providing clear evidence for the ability of Cg3035 to perform the first step in arginine biosynthesis. Interestingly, *N*-acetylglutamine was the only other *N*-acetylated amino acid detectable. This metabolite was previously reported to be produced by strains of the species *C. glutamicum* (formerly “*Brevibacterium lactofermentum”*) [26]. The *N*-acetylglutamine pools were almost identical in all four strains tested (Additional file 4). In addition, relative quantifications of l-citrulline (Additional file 5) and l-arginine (Additional file 6) were performed. Here, the pool of both metabolites significantly decreased in the ∆*cg3035* mutant (fold change 0.16 and 0.50, respectively) and significantly increased about twofold in WT pZ8-1::*cg3035* (fold change 2.10 and 2.28, respectively). For lysine, an amino acid derived from aspartate, neither deletion nor overexpression of *cg3035* had an effect on the intracellular concentration (Additional file 7). Here, it must be mentioned that *cg3035* is not part of the ArgR regulon.

Since *N-*acetylglutamate synthases display a high degree of specificity for acetyl-CoA and l-glutamate as substrates [13], enzyme assays with crude protein extracts were performed to measure the specific NAGS activity of Cg3035. To test for specificity of the NAGS measurement, acetyl-CoA was added as single substrate and no consumption of acetyl-CoA was found (data not shown). Then, NAGS activities were determined in crude extracts of either *C. glutamicum* ATCC 13032 or the deletion mutant ∆*cg3035* carrying the empty overexpression vectors pZ8-1 or pZ8-1::*cg3035* (Table 1). First, a residual NAGS activity in the absence of Cg3035 in the deletion mutant harbouring the empty vector was determined to be 13.8 mU mg^-1^ protein. Second, the endogenous NAGS activity specific for Cg3035 was calculated by subtracting the total NAGS activities of the deletion mutant from that of the wildtype. This was done for strains harbouring the empty vector and for strains with the overexpressed Cg3035 separately. The NAGS activities attributed to Cg3035 were similar at 11.5 mU mg^-1^ protein or 12.9 mU mg^-1^ protein, for vectors pZ8-1 or pZ8-1::*cg3035* respectively. Third, the additional activity conferred by *cg3035* overexpression was 193.7 mU mg^-1^ protein or 206.6 mU mg^-1^ protein, respectively. This is a 16–17 fold increase in activity and can be attributed to the multicopy effect as well as the strong promoter of the expression vector.

**Table 1:** **Specific NAGS activities of *C. glutamicum *** **ATCC 13032 and *C. glutamicum *** **∆** ***cg3035 *** **carrying different plasmids**

**Strain**^**a**^	**Plasmid**	**Specific NAGS activity**	**SD [mU mg**^**-1 **^**protein]**^**b**^
		**[mU mg**^**-1 **^**protein]**	
*C. glutamicum* ATCC 13032	pZ8-1	25.31	0.27
*C. glutamicum* ATCC 13032	pZ8-1::*argJ*	24.59	1.24
*C. glutamicum* ATCC 13032	pZ8-1::*argA*	43.92	3.07
*C. glutamicum* ATCC 13032	pZ8-1::*cg3035*	231.91	9.91
*C. glutamicum* ∆*cg3035*	pZ8-1	13.84	0.30
*C. glutamicum* ∆*cg3035*	pZ8-1::*cg3035*	207.54	8.14

^a^Plasmid harboring cells were cultivated in CGXII medium with kanamycin. ^b^Values are means of triplicate measurements.

In addition, NAGS activities of overexpressed ArgA of *E. coli* and ArgJ of *C. glutamicum* were measured in the wildtype background. As expected, ArgJ exhibited no specific NAGS activity, explaining its inability to complement the *E. coli* ∆*argA* mutant. ArgA from *E. coli* was found to confer additional NAGS activity of approximately 18.6 mU mg^-1^ protein.

For a number of microorganisms, including *E. coli*[27,28], *Salmonella enterica* serovar Typhimurium [29], *Pseudomonas aeruginosa*[30], and *Saccharomyces cerevisiae*[31], a strong feedback inhibition of NAGS activity by l-arginine was found. Therefore, inhibition studies of Cg3035 with l-arginine and also with the intermediate l-ornithine were performed. No changes in specific NAGS activity of the crude extract of *C. glutamicum* ATCC 13032 pZ8-1::*cg3035* were detected when up to 50 mM l-ornithine or l-arginine was added to the reaction mixture (data not shown).

### Cg3035 establishes a novel class of NAGS genes

To determine how related Cg3035 is to other known NAGS genes, comparisons between “classical” ArgA proteins, bifunctional ArgJ, S-NAGS and ArgO from *Campylobacter jejuni*[32] were performed by sequence similarity-based searches with the Cg3035 protein sequence using the basic local alignment search tool (BLAST) [33] at the NCBI. Cg3035 did not show significant overall sequence similarities to members of other classes. Sequence similarity is restricted to the acetyltransferase catalytic domain, being distantly related to that of ArgA from *E. coli* (28% identity in 40 amino acids), ArgJ from *Bacillus subtilis* (29% identity in 41 amino acids) and the S-NAGS form from *M. tuberculosis* (29% identity in 35 amino acids). No such similarity was found to ArgO from *C. jejuni*. Since only the GCN5-related *N*-acetyltransferase (GNAT) domain was found to be conserved, and GNAT-domains can be found in *N*-acetyltransferases belonging to many different functional classes [34], it is apparent that Cg3035 represents a novel NAGS class.

**Figure 5:**
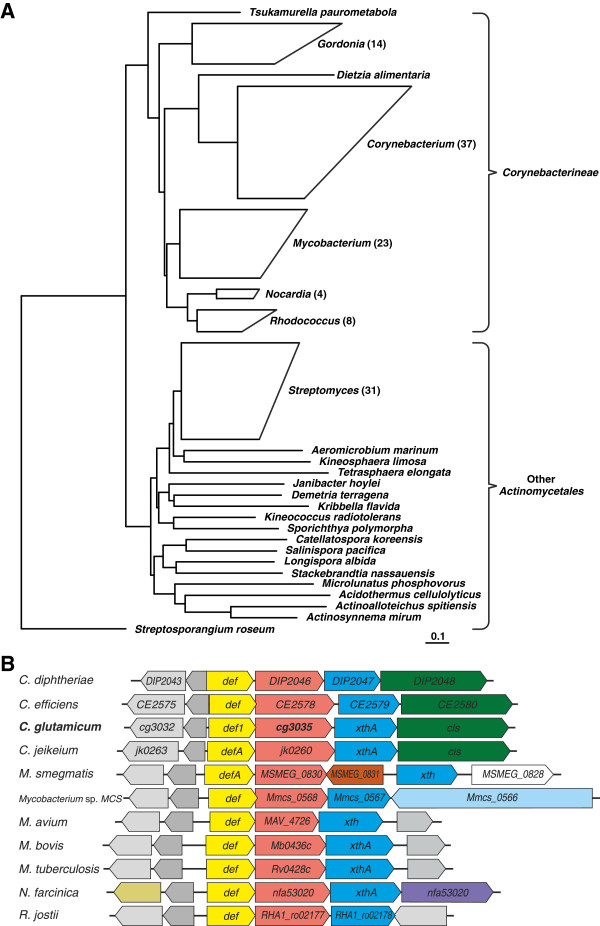
**Phylogenetic analysis of Cg3035 relatives in other organisms and genomic context of *cg3035 *in different bacteria. A)** Cg3035 was used as query against the RefSeq database of BLASTP to identify similar proteins. The most likely orthologues of Cg3035 were aligned using COBALT [35] and a Fast-Minimum-Evolution tree was built with this software. **B)** The freeware tool GeConT II (http://bioinfo.ibt.unam.mx/gecont/index.cgi) was used to visualise the genomic context of *cg3035* and its orthologous genes within fully sequenced bacterial genomes [37]. Species names abbreviated as in http://www.expasy.ch/cgi-bin/speclist.

To identify related sequences and structures of Cg3035, BLASTP [33] with Cg3035 was used as query against the RefSeq database to identify all similar proteins, resulting in 486 hits including the query. The set of results was then filtered retaining only those 383 proteins with at least 25% sequence similarity over 80% of the length of Cg3035. For reasons of clarity, all hits against non-actinobacteria (4) and poorly defined species (72) were excluded. For the same reason, for species with multiple sequenced strains (for example, *M. tuberculosis* or *Corynebacterium diphtheriae*) only one sequence was selected for further analysis, preferably from the type strain, if available. The remaining 138 sequences were aligned using COBALT [35] and a Fast-Minimum-Evolution tree was built (Figure 5a). Based on this tree, it is apparent that there are two distinct groups of possible orthologues: one containing only sequences (88) from the suborder *Corynebacterineae* (“Corynebacteriales”; classification according to latest version of Bergey’s manual [36]) and one comprised of representatives (50) from other suborders of the *Actinomycetales*, mostly *Streptomycineae* (31).

Additional support for these two groups of orthologues can be derived from an analysis of the genomic context: in *Corynebacterineae* (including *Corynebacterium* spp., *Mycobacterium* spp., *Nocardia* spp., *Rhodococcus* ssp. and *Gordonia* ssp.), possible *cg3035* orthologues are strongly associated with *def2*, encoding peptide deformylase, and *xthA*, encoding exodeoxyribonuclease III (Figure 5b). In contrast, the possible orthologue is often located adjacent to a gene encoding a ferredoxin in the second group. Therefore, only the sequences of the most likely orthologues, the 87 from *Corynebacterineae*, were realigned using COBALT and the location of the putative *N*-acyltransferase domain (based on a Pfam-A hit) was marked (Additional file 8).

## Discussion

### Metabolite profiling analyses provide new insight into arginine biosynthesis

In the experiments described here, a systematic approach was performed using defined deletion mutants of *C. glutamicum* that were expected to accumulate intracellular metabolites. No stable isotope labelled internal standards for correction of ion suppression effects were used in these experiments, therefore, only a qualitative analysis of changes in metabolite pools could be performed. Six out of seven intermediates of arginine biosynthesis were found with the applied HPLC-ESI-qTOF method and the results provide new insights concerning metabolism within the arginine pathway. Interestingly, two metabolites accumulated in different mutants – *N*-acetylornithine was found in the two mutants Δ*argRJ* and Δ*argRF,* and citrulline accumulated in Δ*argRG* and Δ*argRH* mutants. In both cases, there was not only an accumulation in the mutant which was defective in the subsequent metabolic reaction, however, also in the strain with an inability to perform the second next reaction. This could indicate a backlog from the accumulated metabolite to its precursor.

*N*-acetylornithine was accumulated in ∆*argRJ*, which confirmed its anticipated function as ornithine acetyl transferase (OAT). The measurements of intracellular *N*-acetylglutamate confirmed this role by demonstrating a strongly reduced pool in *the* ∆*argRJ* mutant. For organisms having such a cyclic arginine pathway, this decrease illustrates a preference for transacetylation by ArgJ instead of using the less economic ArgA activity to produce *N*-acetylglutamate [8].

Since no transacetylation should occur in mutants upstream of ArgJ the behavior of ∆*argRD* mutant is consistent with a low intracellular *N*-acetylglutamate pool. It is puzzling, however, that in the ∆*argRC* mutant the *N*-acetylglutamate pool is similar to the ∆*argRB* mutant and the wildtype. In ∆*argRB* the accumulation of *N*-acetylglutamate can be explained by the interruption of biosynthesis. The unexpected high *N*-acetylglutamate pool in ∆*argRC* can either be interpreted by an equilibrium of the reaction catalysed by ArgB on the side of *N*-acetylglutamate or by a possible instability of the phosphorylated *N*-acetylglutamate and its breakdown to *N*-acetylglutamate. In the wildtype, the feedback inhibition of ArgB is the possible cause for accumulation of *N*-acetylglutamate.

### *In vivo* and *in vitro* experiments confirm the ability of Cg3035 to acetylate l-glutamate

In this study, the function of Cg3035 to use acetyl-CoA for acetylation of glutamate was demonstrated *in vivo* and *in vitro*. Besides complementation of the auxotrophic *E. coli* ∆*argA* mutant, overexpression of *cg3035* led to a higher amount of intracellular *N*-acetylglutamate, whereas deletion of this gene resulted in a reduction of the intracellular *N*-acetylglutamate pool. From *in vitro* enzyme assays using crude extracts it was shown that NAGS enzyme activity also correlated well with the genotype regarding *cg3035*. l-glutamate most probably is the preferred amino acid substrate for Cg3035. Interestingly, the S-NAGS variant in *M. tuberculosis* has a higher activity with l-glutamine [13]. Indeed, *N*-acetylglutamine was found in all *C. glutamicum* strains tested, but in levels unaffected by the presence or absence of *cg3035*. Since no other *N*-acetylated amino acids were found in our metabolome analyses of *C. glutamicum*, this indicates that an involvement of Cg3035 in *N*-acetylglutamine synthesis is unlikely. On the other hand, the correspondence in the pools of *N*-acetylglutamate to those of the amino acids l-citrulline and l-arginine further strengthens the functional assignment of Cg3035 as integral part of arginine biosynthesis.

### Cg3035 – A novel form of *N*-acetylglutamate synthase

The protein encoded by *cg3035* can be compared to known NAGS enzyme classes. Four different kinds of NAGS enzyme families were described until now [2], however, Cg3035 fits into none of them. Different from classical known *N*-acetylglutamate synthases found, for example in *E. coli*, Cg3035 has only a single protein domain. With a length of 335 amino acids, it is shorter than the classical ArgA, although it is larger than the 150–180 amino acids long S-NAGS found, for example, in *M. tuberculosis*.

Homologues of the Cg3035 protein were found in other Corynebacteria as well as in diverse members of the suborder *Corynebacterineae*, such as in *Rhodococcus* spp., *Nocardia* spp., or *Mycobacterium* spp. Analysis of the genomic context revealed a conserved arrangement of three genes encoding peptide deformylase, *N*-acetylglutamate synthase, and exodeoxyribonuclease III among the fully sequenced genomes of this bacterial suborder. Although no functional relationship of these genes is apparent, the conserved genomic context strengthens the view that Cg3035 and its closely similar sequences in the other species of the *Corynebacterineae* represent true orthologues [37].

It is unclear what the evolutionary driving forces were for the formation of the different NAGS protein families. The dissimilarity of NAGS proteins was noted before and attributed to an independent evolution or a more rapid evolution leading to much higher divergence than the sequences of other arginine biosynthesis proteins [1].

Since the growth behaviour experiment on minimal medium revealed a bradytrophy of ∆*cg3035* compared to the wildtype, this indicates that there is at least one other enzyme which is able to perform acetylation of glutamate in *C. glutamicum* ATCC 13032. Candidates for this are proteins with acetyltransferase domains. Besides Cg1722, eight other proteins with acetyltransferase domains are found in *C. glutamicum*, however, these have yet to be characterised.

It could be, however, that Cg3035 is not involved exclusively in arginine biosynthesis. This is not only indicated by the conserved arrangement of *cg3035* with genes involved in peptide or RNA processing. The lack of transcriptional repression of *cg3035* by ArgR and feedback inhibition of Cg3035 by one of the products of arginine biosynthesis, arginine or ornithine also suggests such a role. The initial transacetylation step is a common target to control arginine metabolism, indeed, all previously known microbial NAGS enzymes are feedback-inhibited by arginine. It is well possible that *N*-acetylglutamate is a non-exclusive precursor of arginine biosynthesis, however, is also involved in other biosynthesis pathways. Although it is not known what these biosynthetic pathways produce, the occurrence of *N*-acetylglutamine [38] is an indication for hitherto unknown pathways in Corynebacteria in which *N*-acetylglutamate could play a role. Consequently, in *C. glutamicum*, ArgB would then be the first enzyme of the arginine specific pathway and is feedback-inhibited by l-arginine [10].

## Conclusions

In this study, we investigated the metabolites of arginine biosynthesis in targeted biosynthetic mutants of *C. glutamicum*. Through these investigations, we have found evidence for a missing enzyme which performs the first step of this process, the acetylation of glutamate catalysed by an *N*-acetylglutamate synthase.

By complementation of an auxotrophic *E. coli* ∆*argA* mutant with genomic DNA of *C. glutamicum,* the gene *cg3035* was implicated as the enzyme responsible for this acetylation. A deletion of this gene led to a partial arginine auxotrophy and further experiments on genetic, enzymatic, and metabolomic levels demonstrated the ability of its encoded protein to act as NAGS, using acetyl-CoA for acetylation of glutamate. These findings added significantly to the knowledge on arginine metabolism in *C. glutamicum*. The NAGS enzyme Cg3035 establishes a novel class of *N*-acetylglutamate synthases with orthologues found exclusively in members of the suborder *Corynebacterineae*. Therefore, we propose to name this novel class of NAGS containing Cg3035 and its apparent orthologues C-NAGS (*Corynebacterineae*-type NAGS). This is simply a proposal, and it would be interesting to analyse in future studies if these orthologous candidates demonstrate functional catalysis of the same reaction.

## Methods

### Bacterial strains, growth conditions, plasmids**,** and oligonucleotides

The bacterial strains and overexpression plasmids used in this study are listed in Table 2. Oligonucleotide primers and plasmids for deletions are listed in Additional files 9 and 10. *C. glutamicum* strains were grown at 30°C in CGXII minimal medium (pH 7) consisting of (per liter) 20 g (NH_4_)_2_SO_4_, 5 g urea, 1 g KH_2_PO_4_, 1 g K_2_HPO_4_, 0.25 g MgSO_4_ ∙ 7H_2_O, 10 mg FeSO_4_ ∙ 7H_2_0, 10 mg MnSO_4_ ∙ 7H_2_0, 1 mg ZnSO_4_ ∙ 7H_2_0, 0.2 mg CuSO_4_, 0.02 mg NiCl_2_ ∙ 6H_2_0, 0.2 mg biotin, 0.42 mg thiamine, 0.03 mg protocatechuate and 40 g glucose [39]. The arginine-auxotrophic strains used for metabolic analyses were grown on CASO plates at 30°C for 36 h, inoculated into 10 mL of CGXII medium in 100 mL shake flasks, and incubated o/n at 30°C and 300 rpm. Depending on the cultivated strains and experiments, CGXII media was supplemented with 0.5 mM l-arginine. Main cultures, carried out in 250 mL shake flasks with 20 mL CGXII medium, were inoculated from these precultures to an initial optical density of OD_600_ = 0.4. Cultures were incubated at 30°C and 300 rpm until mid-exponential growth phase (OD_600_ = 5–12); then cells grown without l-arginine were harvested and cells cultivated with supplement were transferred into 50 mL tubes and centrifuged at RT for 5 min at 5,000 × *g.* For depletion of l-arginine, cultures were washed with 20 mL prewarmed (30°C) and unsupplemented CGXII medium. Immediately, cells were resuspended in 20 mL prewarmed CGXII medium without l-arginine, transferred back into sterile 250 mL shake flasks and shaken for another 2 h at 30°C and 300 rpm. For each strain, four biological replicates were prepared. For the generation of ^13^C-labelled internal standard, *C. glutamicum* pZ8-1::*cg3035* was grown in shake flasks with 2% (w/v) [U^13^C]-glucose as sole carbon source.

**Table 2:** Bacterial strains and plasmids

**Name**	**Relevant genotype/information**^**a**^	**Reference/source**
***Strains***		
*E. coli* JM109	F´ *traD36, proA*^*+*^*B*^*+*^*, lacI*^*q*^ Δ*(lacZ,)M15/* Δ*(lac-proAB), glnV44,e14*^*-*^*, gyrA96, recA1, relA1, endA1, thi, hsdR17*	New EnglandBiolabs
*E. coli* JM109 ∆*argA*	JM109 with deleted *argA* gene	This study
*C. glutamicum* ATCC 13032	Wild type (WT), Nx^r^	American type culture collection
∆*argRC*	*C. glutamicum* ATCC 13032 with deleted *argR* and *argC* genes	This study
∆*argRJ*	*C. glutamicum* ATCC 13032 with deleted *argR* and *argJ* genes	This study
∆*argRB*	*C. glutamicum* ATCC 13032 with deleted *argR* and *argB* genes	This study
∆*argRD*	*C. glutamicum* ATCC 13032 with deleted *argR* and *argD* genes	This study
∆*argFR*	*C. glutamicum* ATCC 13032 with deleted *argR* and *argF* genes	[40]
∆*argRG*	*C. glutamicum* ATCC 13032 with deleted *argR* and *argG* genes	This study
∆*argRH*	*C. glutamicum* ATCC 13032 with deleted *argR* and *argH* genes	This study
∆*cg3035*	*C. glutamicum* ATCC 13032 with deleted *cg3035* gene	This study
***Plasmids***		
pK18*mobsacB*	*sacB*, *lacZα*, Km^r^ , mcs mobilizable vector, allows for selection of double crossover in *C. glutamicum*	[41]
pZ8-1	*E. coli-C. glutamicum* shuttle expression vector, P_tac_, Km^r^	[42]
pZ8-2	modified pZ8-1 where the multiple cloning site was exchanged by a sole *Bam*HI-site and a consensus RBS was included	This study
pZ8-1::*argA*	pZ8-1 containing the *argA* structural gene from *E. coli*	This study
pZ8-1::*cg3035*	pZ8-1 containing the *cg3035* structural gene from *C. glutamicum* ATCC 13032	This study
pZ8-1::*argJ*	pZ8-1 containing the *argJ* structural gene from *C. glutamicum* ATCC 13032	This study

^a^r, Superscript indicates resistance; Nx, nalidixic acid; Km, kanamycin.

*E. coli* strains carrying plasmids were routinely grown on solid Antibiotic Medium No. 3 (PA) (Oxoid, Wesel, Germany) at 37°C. Tests for arginine auxotrophy were performed using M9 minimal medium plates [43]. When needed, antibiotics were added at the following concentrations: 50 μg mL^-1^ kanamycin for *E. coli* and 25 μg mL^-1^ kanamycin and 50 μg mL^-1^ nalidixic acid for *C. glutamicum*. Bacterial growth was monitored by measuring the optical density at 600 nm (OD_600_).

### DNA isolation, manipulation, and analysis

Chromosomal DNA of *C. glutamicum* and *E. coli* MG1655 was isolated as described previously [44]. Isolation of plasmid DNA from *E. coli* cells was done by using a GeneJET Plasmid Miniprep Kit (Fermentas, St. Leon-Rot, Germany). PCR amplification of DNA was carried out with Phusion polymerase (Finnzymes, Vantaa, Finland) in an Eppendorf Mastercycler pro S. All PCR setups were done according to the manufacturers´ protocols. Modification of DNA, analysis by agarose gel electrophoresis and ligation were performed using standard procedures [43]. All oligonucleotides used in this study (Additional file 9) were obtained from Metabion (Martinsried, Germany). PCR products were purified with a NucleoSpin Gel and PCR Clean-up Kit (Macherey-Nagel, Düren, Germany). Transformation of *E. coli* with plasmid DNA was performed using the rubidium chloride method [45], *C. glutamicum* cells were transformed by electroporation [46].

### Construction of defined chromosomal deletions

Plasmids for defined chromosomal deletions in *C. glutamicum* ATCC 13032 from start to stop codon were constructed using the geneSOEing method described by Horton *et al.*[47]. In each deletion, the complete coding regions were removed exactly. One exception was the deletion of *argB*, since it contains the ribosome-binding site (RBS) for the *argD* coding sequence located downstream. In this case, the deletion of *argB* was carried out in a way that its RBS was fused to the *argD* coding region in a proper distance. The resulting fusion products were digested with restriction enzymes corresponding to the cleavage sites introduced via PCR (Additional file 9) and ligated into an appropriately digested pK18*mobsacB*[41]. The final non-replicable plasmids were transformed into *C. glutamicum* ATCC 13032 and integration into the chromosome by single-crossover was selected on CASO (Carl Roth, Karlsruhe, Germany) plates supplemented with 25 μg mL^-1^ kanamycin. A second recombination to enable plasmid excision was performed by spreading the transformants on CASO plates containing 10% (w/v) sucrose. Colonies from these plates were tested for the desired kanamycin-sensitive and sucrose-resistant phenotype by parallel picking. PCR experiments were used to verify deletions in the *C. glutamicum* chromosome.

For construction of *E. coli* JM109 ∆*argA* the Quick & Easy *E. coli* Gene Deletion Kit (Gene Bridges, Heidelberg, Germany) was used. Red/ET cloning and removal of the selection marker was performed according to the manufacturer’s instructions.

### Construction of expression vectors

To generate overexpression constructs of genes coding for a putative *N*-acetylglutamate synthase, genes of interest were amplified via PCR using *C. glutamicum* ATCC 13032 or *E. coli* MG1655 genomic DNA, respectively. The PCR primers used are listed in Additional file 9. The resulting PCR products were cleaved using restriction sites added by the PCR primers and ligated into plasmid pZ8-1, digested with the corresponding enzymes. After transformation of *E. coli* JM109, the obtained plasmids were isolated and their inserts sequenced. The final plasmids were then transformed into *E. coli* JM109 ∆*argA* and *C. glutamicum* strains.

To ensure translation of inserted ORFs in *E. coli*, the multiple cloning site of the shuttle expression vector pZ8-1 was exchanged by a sole *Bam*HI-site. Additionally, a consensus ribosomal binding site (RBS) of highly expressed genes (identical to the complement of mRNA-binding site of the 16S rRNA [25]) was cloned upstream this restriction site. The resulting vector was named pZ8-2.

### Generation of a genomic library from *C. glutamicum* ATCC 13032 and complementation of *E. coli* JM109 ∆*argA*

Isolation of genomic DNA was performed as described elsewhere [44]. Five micrograms of DNA were partially fragmented by the restriction enzyme *Bsp*143I (*Sau*3AI) for 4 minutes. Since this step generated sticky ends complementary to the *Bam*HI-site, cloning in a vector was enabled. The size distribution of genomic DNA fragments was analysed on a DNA High Sensitivity chip by an Agilent 2100 Bioanalyzer (Agilent Technologies, Santa Clara, Canada). The total DNA concentration was 41.75 ng μL^-1^, comprising fragments in the range of 700–9000 bp with an average DNA fragment size of 4.2 kb. Genomic DNA fragments were cloned into the constitutively expressing pZ8-2 vector harboring a P_tac_-promoter. Partially digested genome and linear pZ8-2 vector were ligated and transformed into *E. coli* JM109 Δ*argA* mutant. Before plating on M9 minimal medium plates with kanamycin, the transformed cells were washed with 1x TE buffer to remove remaining components of the regeneration medium, for example amino acids. M9 minimal medium plates were incubated for 48 h at 37°C.

### Cell harvesting, metabolite extraction, and sample preparation

Cell harvesting and metabolite extraction were performed as previously [48,49]. Two milliliters of bacterial culture were transferred into a 2 mL reaction tube with screw cap and centrifuged at 20,000 × *g* for 15 s. The supernatant was discarded and the cell pellet was immediately frozen in liquid nitrogen. Frozen cell pellets were freeze-dried within 16 h, using a Christ RVC 2-18 rotational-vacuum-concentrator in combination with a Christ CT 02-50 cooling trap (Martin Christ, Osterode, Germany) and a RZ 2.5 rotary vane pump (Vacuubrand, Wertheim, Germany). A total of 950 mg of silica beads (0.5 mm dia.) and 1 mL of aqueous methanol 80% (v/v) containing 20 μM 2-aminobenzimidazole were added, cells were then disrupted by shaking 3 times in a Precellys 24 (PEQLAB Biotechnologie, Erlangen, Germany) mill at 6,000 rpm with 1 min cycles and 5 s breaks. Cell debris was separated by centrifugation at 20,000 × *g* at RT for 20 min. Seven hundred microliters of the hydrophilic extracts were transferred into 1.5 mL glass vials and evaporated to complete dryness under a nitrogen stream using a Reacti-Therm III heating/stirring module equipped with a Reacti-Vap III nitrogen evaporator (Pierce, Rockford, IL, USA).

### Sample preparation for qualitative LC-MS analysis

Samples were dissolved in 200 μL of water and mixed by vortexing for 10 s. In order to remove remaining particles, samples were transferred into 1.5 mL reaction tubes and centrifuged at 20,000 rpm at 4°C for 20 min. Supernatants were transferred into new 1.5 mL reaction tubes and stored at -80°C. Prior to analysis, a 10 μL aliquot of each sample was transferred into a 1.5 mL glass vial with inlet (5 mm dia.). Subsequently, 0.1 μL formic acid and 90 μL acetonitrile were added. Samples were mixed by vortexing for 5 s and placed into the autosampler.

### Sample preparation for quantitative LC-MS analysis

Samples were dissolved in 50 μL of water and mixed by vortexing for 10 s. Samples were transferred into 1.5 mL reaction tubes and centrifuged at 20,000 rpm at 4°C for 20 min to remove remaining particles. Supernatants were transferred into new 1.5 mL reaction tubes and stored at -80°C. Prior to analysis, a 15 μL aliquot of each sample was transferred into a 1.5 mL glass vial with inlet (5 mm dia.) and 5 μL of ^13^C-labelled internal standard was added. Samples were mixed by vortexing for 5 s and placed into the autosampler.

### HPLC-ESI-QTOF conditions

LC-MS data were obtained using a LaChromUltra (Hitachi Europe, UK) HPLC system coupled to a microTOF-Q hybrid quadrupole/time-of-flight mass spectrometer (Bruker Daltonics, Bremen, Germany), equipped with an electrospray ionisation (ESI) source. Separation of metabolites was performed on a Cogent diamond hydride column (MicroSolv Technologies; 150 × 2.1 mm; 3 μm particles) operated in aqueous normal phase mode. Eluent A: 50% acetonitrile (v/v), 50% water (v/v) + 0.1% (v/v) formic acid and B: 90% acetonitrile (v/v), 10% water (v/v) + 0.1% (v/v) formic acid were prepared freshly and stored in PTFE bottles. Flow rate was set to 400 μL min^-1^ and gradient elution was performed as follows: *t* = 0 min, 100% B; *t* = 6 min 0% B; *t* = 7 min, 0% B; *t* = 8.5 min 100% B; *t* = 13 min 100% B. MS detection was performed with ESI source operated in positive ionisation mode (Additional file 11). Nitrogen was used as sheath, dry, and collision gas. Sodium formate solution (0.1 M) in 50% (v/v) isopropanol was used for external mass calibration and was injected into the ESI source at the beginning of each analysis, using a switch valve and a segmented acquisition method. For targeted detection of *N*-acetylglutamate, multiple reaction monitoring (MRM) was applied. An additional segment was introduced (Additional file 12), during which the pseudomolecular ion was isolated and fragmented to generate characteristic product ions.

### Processing of LC-MS(/MS) Data

Raw data were analysed using the Compass software environment (Bruker Daltonics, Bremen, Germany). Automatic internal mass calibration using an HPC quadratic algorithm and identification of compounds was achieved using Compass DataAnalysis (version 4.0 SP1). The following steps were performed using Compass QuantAnalysis (version 2.0 SP1). This included targeted generation of base peak chromatograms (BPCs) for expected m/z-values of [M + H]^+^ ions (Additional file 13), peak detection, peak integration, and normalisation to the peak areas of the internal standard. Normalisation of resulting peak areas to cell dry weight (CDW) of samples, calculation of mean values, calculation of standard deviation over biological replicates, and relative quantification were performed in a spreadsheet. To test significance of differences in normalised peak areas between different strains, a two-tailed Student’s T-test was applied.

Theoretical fragmentation patterns and mechanisms of selected metabolites were generated on the basis of chemical structure files using Mass Frontier 4.0 Spectral Interpretation Software (HighChem, Bratislava, Slovakia).

### Preparation of crude protein extracts of bacterial cultures

*C. glutamicum* protein extracts were prepared from cells grown to mid-exponential growth phase in liquid CGXII medium. Twenty-five milliliters of bacterial culture was harvested by 10 min of centrifugation at 4°C and 4,500 × *g*. Cell pellets were washed in 25 mL of 100 mM Tris-HCl (pH 7.5) which was supplemented with 20 μM PMSF. After removal of the supernatant, cells were resuspended in 5 mL of 100 mM Tris-HCl (pH 7.5) containing 20 μM PMSF. Cell suspension was split to four 2 mL reaction tubes containing silica beads. Cell disruption using the Precellys 24 bead mill was carried out at a speed ratio of 6.5 for three time intervals of 30 s. Cell debris was removed by 20 min of centrifugation at 4°C and 14,000 rpm. Protein concentrations of the crude extracts were determined by using Roti-Nanoquant (Roth, Karlsruhe, Germany), a modified Bradford assay, according to instructions of the manufacturer.

### Measurement of NAGS enzyme activity

NAGS activity of cell extracts was determined spectrophotometrically with an LKB Biochrom 4060 photometer (Amersham Pharmacia Biotech, Buckinghamshire, England). Increase in absorbance at 412 nm due to formation of 5-thio-2-nitrobenzoate was measured during the reactions (see equation 1 & 2) between the free sulfhydryl group of CoA-SH, generated by the amino acid acetylating activity, and 5,5-dithio-bis(2-nitrobenzoic acid) (DTNB) [13].

(1) $$\begin{array}{l} \text{Acetyl}-\text{CoA}+\text{Glutamate}\overset{\text{enzyme}}{\to }\\ \text{CoA}-\text{SH}+N-\text{Acetylglutamate} \end{array}$$

(2) CoA−SH+DTNB→TNB+CoA−derivative

Reaction was maintained at 25°C and continuously monitored spectrophotometrically in a total volume of 500 μL. Assay mixtures contained 500 mM l-glutamate, 0.2 mM acetyl-CoA and 0.2 mM DTNB. All these substances were dissolved in 100 mM Tris-HCl (pH 7.5). Reactions were initiated by addition of 10 μL of protein extract. One unit of enzymatic activity is the amount of enzyme catalysing the formation of 1 μmol of *N*-acetylglutamate min^-1^ and specific NAGS activity was calculated as the activity of enzyme per milligram of total protein.

## Competing interests

The authors declare that they have no competing interests.

## Authors' contributions

KP constructed the mutants, performed all cloning experiments, enzyme assays, and drafted the manuscript. FW performed and analysed the metabolic profiling experiments. MP participated in the design of the study and worked on the manuscript. CR did the sequence alignments and carried out the phylogenetic analyses. JK conceived the study and finalised the manuscript. All authors read and approved the article.

## Supplementary Material

Additional file 1 **Genes with enhanced or reduced transcription in *C. glutamicum *Δ*argR *compared with *C. glutamicum *ATCC 13032 (reference).** A DNA microarray was performed to compare the transcriptomes of *C. glutamicum* Δ*argR* and *C. glutamicum* ATCC 13032. Total RNA was isolated from two biological replicates grown in minimal CGXII medium to the exponential phase and used for hybridization of two biological and two technical replicates, including a dye-swap. Hybridisation and analysis of the microarray data was carried out as described previously [50,51]. In the table all relevant genes are indicated by their names or locus tags according to the *C. glutamicum* ATCC 13032 genome sequence ([22], GenBank NC_006958). Click here for file

Additional file 2 **Possible ionisation and fragmentation of *N*-acetylglutamate semialdehyde after applying automated MS/MS.** Theoretical fragmentation pattern resulted in the following ions: [M + H]^+^ (m/z = 174.0761), [M + H-H_2_0]^+^ (m/z = 156.0655) and [M + H-H_2_0-C_2_H_2_O]^+^ (m/z = 114.0550), calculated with the software package HighChem Mass Frontier 4.0 (HighChem, Ltd.; Bratislava, Slovakia). Click here for file

Additional file 3 **Growth test with *C. glutamicum *on MM1 minimal medium.** Tenfold serial cell dilutions were prepared and spotted on MM1 minimal medium plates. *C. glutamicum* ATCC 13032 (*Cg* WT) was used as positive control to compare the growth behavior of *Cg* ∆*cg3035.* Click here for file

Additional file 4 **Relative concentrations of *N*-acetylglutamine in different *C. glutamicum *strains.** Hydrophilic metabolites were extracted from *C. glutamicum* ATCC 13032 (WT), ∆*cg3035* as well as WT pZ8-1 (empty vector) and WT pZ8-1::*cg3035.* Peak detection and integration was performed on base peak chromatograms of m/z-values of [M + H]^+^ ions. Error bars represent standard deviations of four biological replicates. An asterisk denotes *p*-values below 0.01. Click here for file

Additional file 5 **Relative concentrations of l-citrulline in different *C. glutamicum *strains.** Hydrophilic metabolites were extracted from *C. glutamicum* ATCC 13032 (WT), ∆*cg3035* as well as WT pZ8-1 (empty vector) and WT pZ8-1::*cg3035.* Peak detection and integration was performed on base peak chromatograms of m/z-values of [M + H]^+^ ions. Error bars represent standard deviations of four biological replicates. An asterisk denotes *p*-values below 0.01. Click here for file

Additional file 6 **Relative concentrations of l-arginine in different *C. glutamicum *strains.** Hydrophilic metabolites were extracted from *C. glutamicum* ATCC 13032 (WT), ∆*cg3035* as well as WT pZ8-1 (empty vector) and WT pZ8-1::*cg3035.* Peak detection and integration was performed on base peak chromatograms of m/z-values of [M + H]^+^ ions. Error bars represent standard deviations of four biological replicates. An asterisk denotes *p*-values below 0.01 and double asterisks mark p-values below 0.05. Click here for file

Additional file 7 **Relative concentrations of l-lysine in different *C. glutamicum *strains.** Hydrophilic metabolites were extracted from *C. glutamicum* ATCC 13032 (WT), ∆*cg3035* as well as WT pZ8-1 (empty vector) and WT pZ8-1::*cg3035.* Peak detection and integration was performed on base peak chromatograms of m/z-values of [M + H]^+^ ions. Error bars represent standard deviations of four biological replicates. An asterisk denotes *p*-values below 0.01. Click here for file

Additional file 8 **Multiple alignment of Cg3035 with its putative orthologues from *Corynebacterineae *species.** A BLASTP search with Cg3035 as query was used against the RefSeq database to identify similar proteins. Only proteins of actinobacteria with at least 25% sequence similarity over 80% length of Cg3035 were used for an alignment using the COBALT [35] software. Click here for file

Additional file 9 **Oligonucleotides used as primers to construct defined *C. glutamicum *** **deletion mutants and expression plasmids.** Click here for file

Additional file 10 Plasmids used for targeted deletions. Click here for file

Additional file 11 Parameters for microTOF control in full scan MS mode. Click here for file

Additional file 12 Additional parameters for microTOF control in MS/MS (MRM) mode. Click here for file

Additional file 13 Relevant parameters of detected compounds. Click here for file
