# Supplementary material for: A novel type of N-acetylglutamate synthase is involved in the first step of arginine biosynthesis in Corynebacterium glutamicum
Source: BMC Genomics. 2013 Oct 18;14:713. doi: 10.1186/1471-2164-14-713 (PMC3827942; doi:10.1186/1471-2164-14-713)
Supplement: Additional file 1 — Genes with enhanced or reduced transcription in C. glutamicum ΔargR compared with C. glutamicum ATCC 13032 (reference). A DNA microarray was performed to compare the transcriptomes of C. glutamicum ΔargR and C. glutamicum ATCC 13032. Total RNA was isolated from two biological replicates grown in minimal CGXII medium to the exponential phase and used for hybridization of two biological and two technical replicates, including a dye-swap. Hybridisation and analysis of the microarray data was carried out as described previously [50,51]. In the table all relevant genes are indicated by their names or locus tags according to the C. glutamicum ATCC 13032 genome sequence ([22], GenBank NC_006958). [file 1471-2164-14-713-S1.pdf]

Additional file 1:

Additional method: Microarray hybridization

The hybridization of whole-genome oligonucleotide microarrays was performed as described previously [48], using 10 µg of total RNA from *C. glutamicum* cultures for cDNA synthesis. The normalization and evaluation of the hybridization data was done with the software package EMMA 2 [49] using a signal intensity (A-value) cut-off of ≥7.0 and a signal intensity ratio (M-value) cut-off of ±0.6 (corresponding to fold changes of 1.52 and 0.66, respectively).

Comparison of gene expression in *C. glutamicum* ATCC 13032 vs. Δ*argR*

Coding sequence	Gene	Fold change[a]	Annotation
cg1580	*argC*	9.45	N-acetyl-γ-glutamyl-phosphate reductase
cg1584	*argF*	9.13	Ornithine carbamoyltransferase
cg1583	*argD*	9.06	Acetylornithine aminotransferase, AT class II
cg1581	*argJ*	8.34	Glutamate N-acetyltransferase
cg1582	*argB*	7.62	Acetylglutamate kinase
cg1586	*argG*	6.63	Argininosuccinate synthase
cg1588	*argH*	5.82	Argininosuccinate lyase
cg1813	*carB*	5.17	Carbamoyl-phosphate synthase, large chain
cg1812	*pyrF*	4.06	Orotidine-5'-phosphate decarboxylase
cg1814	*carA*	3.94	Carbamoyl-phosphate synthase, small chain
cg0536		3.81	Putative 5-dehydro-4-deoxyglucarate dehydratase
cg0535		3.39	Putative ketoglutarate semialdehyde dehydrogenase
cg0534		2.91	Putative integral membrane protein
cg3226		2.43	Putative MFS-type L-lactate permease
cg3145		2.36	Hypothetical protein
cg2636	*catA*	2.33	Catechol 1.2-dioxygenase
cg0611		2.25	Putative secreted protein
cg3227	*lldD*	2.17	Quinone dependent L-lactate dehydrogenase
cg1589		1.95	Putative secreted protein
cg0749	*spoU*	1.87	Putative tRNA/rRNA methyltransferase

cg1737	*acn*	1.82	Aconitate hydratase
cg0607		1.77	Putative secreted protein
cg3431	*rnpA*	1.74	Ribonuclease P
cg2546		1.73	Putative secondary C4-dicarboxylate transporter, TRAP-T family
cg0446	*sdhA*	1.69	Succinate dehydrogenase, subunit A
cg1695		1.67	Conserved hypothetical protein, probably HTH-type transcriptional reg.
cg0228		1.65	Two-component system, sensory histidine kinase, putative pseudogene
cg2320		1.64	Putative transcriptional regulator, ArsR-family
cg1343	*narH*	1.62	Respiratory nitrate reductase 2, beta chain
cg2235	*rplS*	1.61	50S ribosomal protein L19
cg2348		1.60	Putative secreted protein
cg1090	*ggtB*	1.58	Putative gamma-glutamyltranspeptidase
cg0445	*sdhCD*	1.57	Succinate dehydrogenase, subunit CD
cg1546	*rbsK1*	1.57	Putative ribokinase
cg2751		1.57	Xanthosine triphosphate pyrophosphatase
cg0961		1.54	Putative hydrolase, alpha/beta-fold
cg2402		1.54	Secreted protein NLP/P60 family
cg2574		1.54	Put. threonine efflux transporter, resistance to homoserine/threonine (RhtB) family
cg1680		1.53	Short-chain dehydrogenase/reductase
cg2071	*int2'*	1.53	Putative phage Integrase (N-terminal fragment)
cg0447	*sdhB*	1.52	Succinate dehydrogenase, subunit B
cg1279		0.66	Putative secreted protein
cg2199	*pbp*	0.66	Putative penicillin-binding protein
cg2824		0.66	SAM-dependent methyltransferase
cg0009		0.65	Putative membrane protein
cg0365		0.65	Putative membrane protein
cg0926		0.65	ABC-type putative iron-siderophore transporter, permease subunit
cg0119	*ureD*	0.65	Urease accessory protein UreD
cg1405		0.64	Siderophore-interacting protein
cg2777		0.63	Conserved putative membrane protein
cg2052		0.63	Putative secreted protein
cg0924		0.62	ABC-type putative iron-siderophore transporter, substrate-binding lipoprotein
cg2962		0.62	Hypothetical protein
cg0726		0.61	Putative secreted lipoprotein

cg1120	*ripA*	0.60	Putative transcriptional regulator, AraC-family
cg2950	*radA*	0.60	Putative ATP-dependent protease, involved in DNA repair
cg0767		0.58	Siderophore-interacting protein
cg2283		0.58	Conserved hypothetical protein
cg0927		0.58	ABC-type putative iron-siderophore transporter, permease subunit
cg1931		0.57	Putative secreted protein
cg1476	*thiC*	0.57	Thiamine biosynthesis protein ThiC
cg0748		0.57	ABC-type putative iron-siderophore transporter, substrate-binding lipoprotein
cg1930		0.54	Putative secreted hydrolase
cg0922		0.52	ABC-type putative iron-siderophore transporter, substrate-binding lipoprotein
cg0771	*irp1*	0.50	ABC-type putative iron-siderophore transporter, substrate-binding lipoprotein
cg4005		0.50	Putative secreted protein
cg1514		0.49	Secreted protein
cg1412	*rbsC*	0.39	ABC-type ribose transporter, permease subunit (TC 3.A.1.2.1)
cg0405		0.38	ABC-type putative iron(III) dicitrate transporter, substrate-binding lipoprotein
cg1411	*rbsA*	0.38	ABC-type ribose transporter, ATPase subunit (TC 3.A.1.2.1)
cg0471		0.37	Conserved secreted protein
cg1413	*rbsB*	0.25	ABC-type ribose transporter, substrate-binding lipoprotein (TC 3.A.1.2.1)
cg1585	*argR*	0.16	Transcriptional repressor of arginine biosynthesis, ArgR-family

All genes that showed an alteration of transcript levels at least by a factor of ±2 in two independent transcriptome analyses (biological replicates) using DNA microarrays with fourfold genome coverage are listed.

[a] Fold change, signal intensity ratio as defined by $2^{(m\text{-}value)}$.
