# Supplementary material for: A novel type of N-acetylglutamate synthase is involved in the first step of arginine biosynthesis in Corynebacterium glutamicum
Source: BMC Genomics. 2013 Oct 18;14:713. doi: 10.1186/1471-2164-14-713 (PMC3827942; doi:10.1186/1471-2164-14-713)
Supplement: Additional file 9 — Oligonucleotides used as primers to construct defined C. glutamicum deletion mutants and expression plasmids. [file 1471-2164-14-713-S9.pdf]

Additional file 9: Oligonucleotides used as primers to construct defined *C. glutamicum* deletion mutants and expression plasmids

**Name**	**Sequence** [a]	**Product**
*argR*_d1	GATCTAGAATTCTCGTGCGGAATTCGTGGA	Δ*argR*
*argR*_d2	*TTACCTGTGAGCGGATCA*TGTACCTGGCTGGTGACT	
*argR*_d3	*TGATCCGCTCACAGGTAA*	
*argR*_d4	GATCTAGGATCCCTCAACGAGGTGCTTGAC	
*argC*_d1	GGTGGTCTCGAATTACCGTAAGCTTGCGGCTTCC	Δ*argC*
*argC*_d2	*CGCCGCTTTAAGGCGTACT*TGCAAATTATTCATGCATAAA	
*argC*_d3	*AGTAGCGCCTTAAAGCGGCG*	
*argC*_d4	GGTGGTCTCGGATCCTGCACCATTGTCACCCAAA	
*argJ*_d1	GGTCAATTGCGCCACACCCCGGAAATTGC	Δ*argJ*
*argJ*_d2	*AGTTGGAGTGCTGTTTCTTT*TGTGGAACTCCTACTGAATT	
*argJ*_d3	*AAAGAAACAGCACTCCAACT*	
*argJ*_d4	GGTAGATCTAGTGGGTACGCCATCGATGT	
*argB*_d1	GGTGGTCTCGAATTCAACTCAGGATGAACTCAACG	Δ*argB*
*argB*_d2	*CAAGTTTCCAGCGTGCTCAT*TGCCTGTGCCCTTTTCCCTG	
*argB*_d3	*ATGAGCACGCTGGAAACTTG*	
*argB*_d4	GGTGGTCTCGGATCGGGTAGAACTCCACACCGCT	
*argD*_d1	GGTGAATTCTTGGTTTGGTCGGAGACATC	Δ*argD*
*argD*_d2	*AAGTCATAAGTTTGAGTCCT*TTTACAGTTCCCCATCCTTG	
*argD*_d3	*AGGACTCAAACTTATGACTT*	
*argD*_d4	GGTAGATCTCAGCCTTCTTACCCTTAAGG	
*argRG*_d1	GGTGGTCTCGAATTCAGGCCTTAAGGGTAAGAAG	Δ*argRG*
*argRG*_d2	*TGGATGCTTGAAAAGGTGGC*GTCTTACCTCGGCTGGTTGG	
*argRG*_d3	*GCCACCTTTTCAAGCATCCA*	
*argRG*_d4	GGTGGTCTCGGATCGAGGGCGTCGACAAGCTCGG	
*argH*_d1	GGTGGTCTCGAATTTCAAGTCCCGCGAAATCTAC	Δ*argH*
*argH*_d2	*AAGCAGCACAGGCCATAAAC*GTTCCATGTGGTGTTCTTCT	
*argH*_d3	*GTTTATGGCCTGTGCTGCTT*	
*argH*_d4	GGTGGTCTCGGATCTCGGTGATGGTTCCGAATGT	
*cg3035*_d1	GGTGGTCAATTGGAAATCCCAGAGACCATGCC	Δ*cg3035*
*cg3035*_d2	*TACCCTAGCTGTTTTGGATT*TCCGCATCAGACGGCATCCC	
*cg3035*_d3	*AATCCAAAACAGCTAGGGTA*	
*cg3035*_d4	GGTGGTAGATCTTGAAGTCGCCGAGCAACACC	
*argA*_RedET1	AACAGAATAAAAATACACTAATTTCGAATAATCATGCAAA - GAGGTGTGCC*AACCCTCACTAAAGGGCGGC*	Δ*argA*
*argA*_RedET2	GGATGCGACGTACGAGTGTTACGCATGTCGCATCCGAC-GATTTTCATCGC*CGACTCACTATAGGGCTCGA*	
*argA*_e1	AGGTCTCGAATTG**AAAGGAGGACAACC**GTGGTAAAGG-AACGTAAAAC	*argA*
*argA*_e2	AGGTCTCGGATCGTTACCCTAAATCCGCCATCA	
*argJ*_e1	GGGTCTCTAATT**AAAGGAGGACAACC**ATATGGCAGAAA-AAGGCATTAC	*argJ*
*argJ*_e2	GGTCTCCTCGATTACCCGGGAGAGCTGTACGCGGAGTTGA	
*cg3035*_e1	AGGTCTCGAATTG**AAAGGAGGACAACC**ATGACGCCTAG-TCTTCCCCG	*cg3035*
*cg3035*_e2	AGGTCTCGGATCGTTAGAATTTCCGTTCGGCGT	

[a] Underlines indicate restriction sites added for cloning. Italics denotes annealing regions for gene SOEing PCR primers or in case of the RedET recombination system overlaps for the cassette. The artificial RBS is given in boldface.
