# Supplementary material for: A novel type of N-acetylglutamate synthase is involved in the first step of arginine biosynthesis in Corynebacterium glutamicum
Source: BMC Genomics. 2013 Oct 18;14:713. doi: 10.1186/1471-2164-14-713 (PMC3827942; doi:10.1186/1471-2164-14-713)
Supplement: Additional file 10 — Plasmids used for targeted deletions. [file 1471-2164-14-713-S10.pdf]

Additional file 10: Plasmids used for targeted deletions

**Name**	**Relevant genotype/ information**	**Reference/ source**
pK18::Δ*argR*	pK18*mobsacB* with a 346 bp deletion construct of gene *argR*	This study
pK18::Δ*argC*	pK18*mobsacB* with a 1044 bp deletion construct of gene *argC*	This study
pK18::Δ*argJ*	pK18*mobsacB* with a 1166 bp deletion construct of gene *argJ*	This study
pK18::Δ*argB*	pK18*mobsacB* with a 953 bp deletion construct of gene *argB*	This study
pK18::Δ*argD*	pK18*mobsacB* with a 1175 bp deletion construct of gene *argD*	This study
pK18::Δ*argFR*	pK18*mobsacB* with a deletion construct of genes *argFR*	[48]
pK18::Δ*argRG*	pK18*mobsacB* with a 1889 bp deletion construct of genes *argRG*	This study
pK18::Δ*argH*	pK18*mobsacB* with a 1434 bp deletion construct of gene *argH*	This study
pK18::Δ*cg3035*	pK18*mobsacB* with a 829 bp deletion construct of gene *cg3035*	This study
