# Supplementary material for: A novel type of N-acetylglutamate synthase is involved in the first step of arginine biosynthesis in Corynebacterium glutamicum
Source: BMC Genomics. 2013 Oct 18;14:713. doi: 10.1186/1471-2164-14-713 (PMC3827942; doi:10.1186/1471-2164-14-713)
Supplement: Additional file 11 — Parameters for microTOF control in full scan MS mode. [file 1471-2164-14-713-S11.pdf]

Additional file 11: Parameters for microTOF control in full scan MS mode

**Mode**	**Scan Mode**	MS	**Ion Polarity**	Positive
	**Mass Range**	50-1000 m/z	**Rolling Average**	off
	**Spectra Acquisition**	Save Spectra	**Absolute Threshold**	10
	**Include Profile Spectra**	Always	**Peak Summation Width**	5 pts
	**Focus**	Inactive	**Acquisition Rate**	1.0 Hz
**Source**	**Endplate Offset**	-500 V	**Dry Gas**	8.0 L min^{-1}
	**Capillary**	-2500 V	**Dry Temp**	180 °C
	**Nebulizer**	3.0 bar		
**Transfer**	**Funnel 1 RF**	180.0 Vpp	**ISCID Energy**	0.0 eV
	**Funnel 2 RF**	200.0 Vpp	**Hexapole RF**	100.0 Vpp
**Quadrupole**	**Ion Energy**	5.0 eV	**Low Mass**	100.00 m/z
**Collision Cell**	**Collision Energy**	10.0 eV	**Collision RF**	150.0 Vpp
	**Transfer Time**	70.0 µs	**Pre Puls Storage**	7.0 µs
