# Supplementary material for: A novel type of N-acetylglutamate synthase is involved in the first step of arginine biosynthesis in Corynebacterium glutamicum
Source: BMC Genomics. 2013 Oct 18;14:713. doi: 10.1186/1471-2164-14-713 (PMC3827942; doi:10.1186/1471-2164-14-713)
Supplement: Additional file 12 — Additional parameters for microTOF control in MS/MS (MRM) mode. [file 1471-2164-14-713-S12.pdf]

Additional file 12: Additional parameters for microTOF control in MS/MS (MRM) mode

Time Segment	Isol. Mass	Isol. Width	Coll. Energy	ISCID Energy	Acq. Factor
0.5 -2.5 min	190.10	10.00	20.00	0.0	1.0
