# Supplementary material for: A novel type of N-acetylglutamate synthase is involved in the first step of arginine biosynthesis in Corynebacterium glutamicum
Source: BMC Genomics. 2013 Oct 18;14:713. doi: 10.1186/1471-2164-14-713 (PMC3827942; doi:10.1186/1471-2164-14-713)
Supplement: Additional file 13 — Relevant parameters of detected compounds. [file 1471-2164-14-713-S13.pdf]

Additional file 13: Relevant parameters of detected compounds

**Compound**	**Sum Formula**	**Exact Mass [g/mol]**	**Monitored Ion(s)**	**BPC m/z**	**Ret. Time [min]**
2-aminobenzimidazole (ISTD)	$C_7H_7N_3$	133.0640	$[M+H]^+$	134. 0713	5.4
N-acetylglutamate	$C_7H_{11}NO_5$	189.1659	$[M+H]^+$	190.1729	1.5
N-acetylglutamyl phosphate	$C_7H_{12}NO_8P$	269.0301	$[M+H]^+$	270.0373	n.d.
N-acetylglutamate semialdehyde	$C_7H_{11}NO_4$	173.0688	$[M+H]^+$	174.0761	1.6
N-acetylornithine	$C_7H_{14}N_2O_3$	174.1004	$[M+H]^+$	175.1074	6.4
L-ornithine	$C_5H_{12}N_2O_2$	132.0899	$[M+H]^+$	133.0972	8.0
L-citrulline	$C_6H_{13}N_3O_3$	175.0957	$[M+H]^+$	176.1027	6.2
$[U^{13}C]$- L-citrulline	$^{13}C_6H_{13}N_3O_3$	181.1160	$[M+H]^+$	182.1230	6.2
L-argininosuccinate	$C_{10}H_{18}N_4O_6$	290.1226	$[M+H]^+$	291.1296	7.3
L-arginine	$C_6H_{14}N_4O_2$	174.1117	$[M+H]^+$	175.1190	8.0
$[U^{13}C]$-L-arginine	$^{13}C_6H_{14}N_4O_2$	180.1349	$[M+H]^+$	181.1393	8.0
N-acetylglutamine	$C_7H_{12}N_3O_4$	188.0797	$[M+H]^+$	189.087	2.3
$[U^{13}C]$-*N*-acetylglutamine	$^{13}C_7H_{12}N_3O_4$	195.1032	$[M+H]^+$	196.116	2.3
L-lysine	$C_6H_{14}N_2O_2$	146.1055	$[M+H]^+$	147.1128	8.1
$[U^{13}C]$- L-lysine	$^{13}C_6H_{14}N_2O_2$	152.1258	$[M+H]^+$	153.1331	8.1
N-acetylglutamate (MRM mode)	$C_7H_{11}NO_5$	189.1659	Fragments	84.044 +102.056 +130.048 +148.050	1.5
$[U^{13}C]$-*N*-acetylglutamate (MRM mode)	$^{13}C_7H_{11}NO_5$	196.1894	Fragments	88.055 +106.068 +135.065 +153.073	1.5

BPC: Base Peak Chromatogram; n.d.: not determined
